# A Cascade Signal Amplification Strategy for the Ultrasensitive Fluorescence Detection of Cu^2+^ via λ-Exonuclease-Assisted Target Recycling with Mismatched Catalytic Hairpin Assembly

**DOI:** 10.3390/bios13100918

**Published:** 2023-10-08

**Authors:** Zhen Liu, Chen Liu, Liqiong He, Jinquan Liu, Le Li, Shengyuan Yang, Yan Tan, Xing Liu, Xilin Xiao

**Affiliations:** 1 Hunan Key Laboratory of Typical Environmental Pollution and Health Hazards, School of Public Health, Hengyang Medical School, University of South China, Hengyang 421001, China; 20212014211130@stu.usc.edu.cn (Z.L.); hlq_joan@163.com (L.H.); usclile@126.com (L.L.); yangshyhy@126.com (S.Y.); tanyan007@163.com (Y.T.); 20222014111306@stu.usc.edu.cn (X.L.); 2 Hunan Province Key Laboratory for Typical Environmental Pollution and Health Hazards, School of Chemistry and Chemical Engineering, University of South China, Hengyang 421001, China; liuchen5573@email.swu.edu.cn; 3 State Key Laboratory of Chemo & Biosensing and Chemometrics, Hunan University, Changsha 410082, China

**Keywords:** Cu^2+^ detection, λ-exonuclease-assisted target recycling, mismatched catalytic hairpin assembly, cascade signal amplification, environmental pollutant

## Abstract

Herein, an ultrasensitive DNAzyme-based fluorescence biosensor for detecting Cu^2+^ was designed using the cascade signal amplification strategy, coupling λ-exonuclease-assisted target recycling and mismatched catalytic hairpin assembly (MCHA). In the designed detection system, the target, Cu^2+^, can activate the Cu^2+^-dependent DNAzyme to cause a cleavage reaction, releasing ssDNA (*t*DNA). Then, *t*DNA binds to hairpin DNA (H0) with an overhanging 5′-phosphorylated terminus to form dsDNA with a blunt 5′-phosphorylated terminus, which activates the dsDNA to be digested by λ-Exo and releases *t*DNA along with another ssDNA (*i*DNA). Subsequently, the *i*DNA initiates MCHA, which can restore the fluorescence of carboxyfluorescein (FAM) previously quenched by tetramethylrhodamine (TAMRA), resulting in a strong fluorescent signal. Furthermore, MCHA efficiently improves the signal-to-noise ratio of the detection system. More importantly, *t*DNA recycling can be achieved with the λ-Exo digestion reaction to release more *i*DNA, efficiently amplifying the fluorescent signal and further improving the sensitivity to Cu^2+^ with a detection limit of 60 *f*M. The practical application of the developed biosensor was also demonstrated by detecting Cu^2+^ in real samples, proving it to be an excellent analytical strategy for the ultrasensitive quantification of heavy metal ions in environmental water sources.

## 1. Introduction

Copper (Cu), as one of the transition metals necessary for the human body, plays a critical role in various basic physiological processes involving metalloenzymes [1,2,3]. However, high concentrations of copper (II) ions (Cu^2+^) may cause serious damage to the human nervous system and liver, potentially causing conditions such as Alzheimer’s disease [4] and Wilson’s disease [5]. Hence, it is imperative for human health to develop a standard and sensitive analytical strategy for monitoring Cu^2+^ [6]. Traditional analytical strategies, such as atomic absorption spectroscopy (AAS) [7], inductively coupled plasma–mass spectrometry (ICP-MS) [8], and surface-enhanced Raman spectroscopy (SERS) [9], have been extensively used as standard procedures for detecting Cu^2+^ in water samples. Nevertheless, these sensitive strategies face certain barriers, such as undesirable costs and a cumbersome procedure. Therefore, it is vital to realize a more practical strategy for monitoring Cu^2+^.

Isothermal amplification strategies, comparable to the polymerase chain reaction (PCR), which serves as the gold standard in amplification strategies for nucleic acid testing [10], are often used for signal amplification in biosensors [11,12]. Isothermal amplification strategies are based on a nucleic acid reaction involving the hydrolysis of nucleic acids [13,14], the replication of nucleic acids [15,16], and nucleic acid recycling [17,18]. Depending on whether nucleases are used or not, isothermal amplification strategies are divided into enzyme-assisted [19,20] and enzyme-free strategies [21,22]. As a highly processive 5′–3′ dsDNA exonuclease, λ exonuclease (λ-Exo) can progressively digest a phosphorylated strand from the 5′-end to the 3′-end, while exhibiting hardly any activity toward non-phosphorylated dsDNA, thereby generating ssDNA and mononucleotides [23,24]. Hence, λ-Exo is an effective nuclease for recycling the amplification of a target sequence [25,26]. On the other hand, catalytic hairpin assembly (CHA), as a representative enzyme-free amplification strategy [27], is a stabilized circulating strand displacement reaction utilizing metastable DNA hairpins [28,29]. In conventional CHA, a pair of DNA hairpins are designed to be partially complementary to each other, which is thermodynamically favorable but kinetically blocked for the structure of DNA hairpins [30]. Upon the introduction of catalytic ssDNA (initiator), the first hairpin DNA (H1) reacts and becomes unblocked through a toehold-mediated strand displacement reaction, exposing a new toehold region. Then, this toehold strand equivalent to the initiator reacts with and opens the second hairpin DNA (H2), releasing the initiator and forming an H1/H2 complex. Then, the released initiator is recycled, triggering a new round of the nucleic acid circuit. In fact, the helix ends in the hairpin substrates are more likely to “breathe” than internal base pairs, leading to a non-negligible background leakage. In order to decrease the background leakage, the Ellington group first introduced mismatched base pairs into the active breathing site of H2, improving the signal-to-noise ratio (S/N) [31]. Although the above two strategies have a clear effect, single amplification strategies cannot enable the detection of a target at ultralow concentrations. Therefore, strategies involving cascade amplification have been introduced.

Herein, an ultrasensitive DNAzyme-based fluorescence biosensor for detecting Cu^2+^ is designed with a cascade signal amplification strategy combining λ-exonuclease-assisted target recycling and MCHA. There are three main parts of the reaction in the biosensor. First, the Cu^2+^-dependent DNAzyme is activated by Cu^2+^ and specifically catalyzes the cleavage of S-DNA through E-DNA, releasing ssDNA (*t*DNA). Second, hairpin DNA (H0) with an overhanging 5′-phosphorylated terminus can bind to the released ssDNA, forming dsDNA with a blunt 5′-phosphorylated terminus. The dsDNA with a blunt 5′-phosphorylated terminus is progressively digested by λ-Exo, thus releasing *t*DNA and *i*DNA (initiator). The released *t*DNA can be further used in later cycles of the hybridization and digestion procedure (recycle I), generating substantial amounts of *i*DNA. Lastly, equipped with H1 and H2, MCHA is triggered by this *i*DNA (recycle II). Via the recovery of FAM fluorescence previously quenched by TAMRA, the circuit system emits a strong fluorescence signal. Benefiting from MCHA, the S/N ratio of the cascade signal amplification strategy is effectively maximized. More importantly, *t*DNA recycling can be achieved with the λ-Exo digestion reaction to release more *i*DNA, efficiently amplifying the fluorescent signal and further improving the sensitivity to Cu^2+^. The proposed biosensor can provide an excellent analytical strategy for the ultrasensitive quantification of heavy metal ions in environmental water sources.

## 2. Materials and Methods

### 2.1. Materials and Reagents

Cu(NO_3_)_2_, Na_2_HPO_4_, sodium ascorbate, boric acid, 1,4-dithiothreitol (DTT), and ethylenediaminetetraacetic acid disodium salt were purchased from Aladdin Reagent Co., Ltd. (Shanghai, China). Tris(hydroxymethyl) aminomethane, NaCl, and MgCl_2_ were bought from Sinopharm Group Co., Ltd. (Shanghai, China). HCl was bought from Hengyang Kaixin Chemical Reagent Co., Ltd. (Hengyang, China). SYBR Green I and DNA Marker were bought from Sangon Biotechnology Co., Ltd. (Shanghai, China). λ-Exo was obtained from New England Biolabs. Ltd. (Beijing, China). UO_2_(NO_3_)_2_, Pb(NO_3_)_2_, HgCl_2_, AgNO_3_, CrCl_3_, AlCl_3_, FeCl_3_, and KCl were supplied by Sinopharm Chemical Reagent Co., Ltd. (Shanghai, China). All the conventional chemical reagents were of analytical grade. The DNA oligonucleotides used were ordered from Sangon Biotechnology Co., Ltd. (Shanghai, China) (Table S1).

The types of buffers used were as follows: buffer A (50 mM Na_2_HPO_4_ and 500 mM NaCl; pH 7.6), buffer B (50 mM Na_2_HPO_4_ and 50 mM NaCl; pH 7.6), buffer C (25 mM Tris-HCl, 50 mM NaCl, 2 mM MgCl_2_, and 1 mM DTT; pH 9.4), and buffer D (50 mM Na_2_HPO_4_ and 1 mM EDTA; pH 7.6). Among these, buffer A was employed as the DNA stock solution (10.0 μmol·L^−1^) of oligonucleotides, while the others were used as DNA reaction buffers. The solvent in each step was deionized water (DI water, 18 MΩ·cm) from a Milli-Q water purification system.

### 2.2. Apparatus

The fluorescence spectrum was obtained by using an FL 7000 fluorescence spectrometer (Hitachi Co., Beijing, China). The fluorescence spectrometer parameters are listed in the Supplementary Materials.

Polyacrylamide gel electrophoresis (PAGE) analysis experiments were carried out on a gel electrophoresis instrument (Bio-Rad, Guangzhou, China). The gel was imaged using a ChemiDox XRS imaging system, and the experimental results were analyzed using Image Lab (Bio-Rad, Guangzhou, China). The PAGE process parameters are recorded in the Supplementary Materials.

### 2.3. Procedure for Cu^2+^ Detection

At first, 9 μL of Cu^2+^-dependent DNAzyme (5.0 μmol·L^−1^), 3 μL of Cu^2+^ at various concentrations, 2.5 μL of ascorbic acid (5.0 mmol·L^−1^), and 35.5 μL of buffer B were added to a PE tube and incubated at 25 °C for 30 min, which resulted in DNAzyme cleavage. Subsequently, 20 μL of the abovementioned solution was mixed with 3 μL of H0 (10.0 μmol·L^−1^) in another EP tube containing 46 μL of buffer C, which was incubated at 25 °C for 30 min. Then, 6 μL of λ-Exo (2 U/μL) was further added, before incubating at 37 °C for 1 h. After digestion, the mixture was heated to 80 °C for 10 min to inactivate the λ-Exo. After the temperature returned to room temperature, 3 μL of H1, 3 μL of H2, and 219 μL of buffer D (50 mM Na_2_HPO_4_ and 1 mM EDTA; pH 7.6) were successively added to the mixture, before incubating at 25 °C in the dark for 2 h.

In this experiment, we used the ratio signal (F/F_0_) as a guideline, where F and F_0_ represent the fluorescence intensity in the presence and absence of the target (Cu^2+^), respectively.

### 2.4. Detection of Cu^2+^ in Real Samples

The collected water as a real sample was filtered with a 0.22 μm filter membrane, and the pH value was adjusted to 7.6 before testing. The actual water availability of the constructed biosensor was assessed by measuring the original and spiked samples.

## 3. Results and Discussion

### 3.1. Principle of the Designed Cu^2+^ Fluorescence Biosensor

**Scheme 1:**
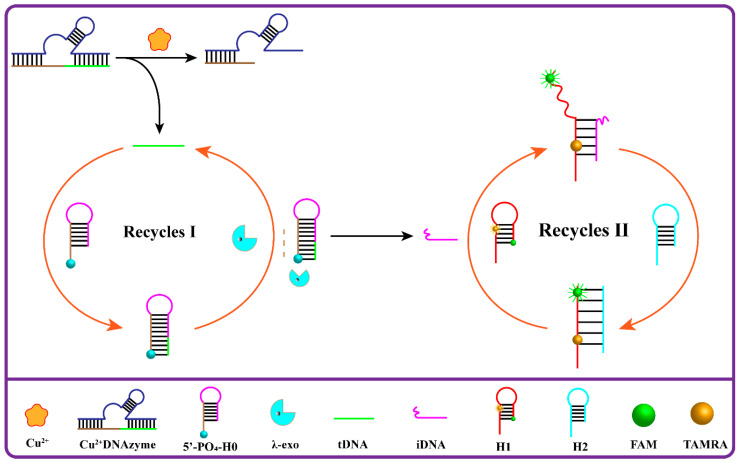
Schematic diagram of the Cu^2+^ fluorescence biosensor with cascade signal amplification.

As shown in Scheme 1, the ultrasensitive DNAzyme-based fluorescence biosensor for detecting Cu^2+^ was designed using a cascade signal amplification strategy coupling λ-exonuclease-assisted target recycling and MCHA. In this method, in the absence of Cu^2+^, a Cu^2+^-dependent DNAzyme containing a sequence fragment that could trigger “recycle I” coexists concordantly with DNAs with a rigid structure including H0, H1, and H2. Importantly, the base mismatch between H1 and H2 can effectively minimize the background signal of the assay. However, with the addition of Cu^2+^, the Cu^2+^-dependent DNAzyme is activated and catalyzes the cleavage of S-DNA, releasing ssDNA (tDNA). Then, the released tDNA binds to H0 to form a new hairpin DNA with a blunt 5′-phosphorylated terminus, which triggers λ-exonuclease-assisted target recycling and then releases tDNA and iDNA. Subsequently, the released iDNA acts as an initiator to open H1 and initiate MCHA, while the released tDNA hybridizes with the remaining H0 to realize “recycle I”. At the same time, “recycle II” is initiated. Then, the fluorescence of FAM is restored, generating a powerful fluorescent signal and thus achieving the ultrasensitive detection of Cu^2+^.

### 3.2. Feasibility of the Fluorescence Biosensor for Cu^2+^ Detection

**Figure 1:**
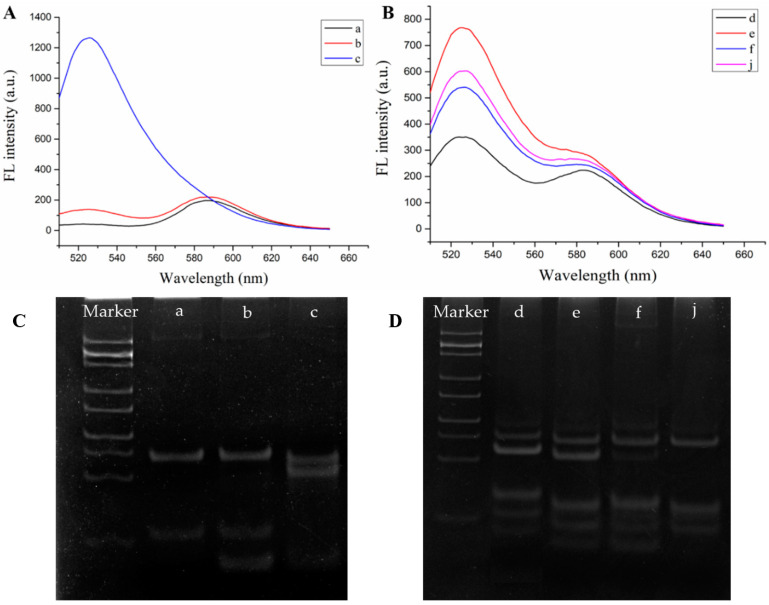
Fluorescence responses of the system (**A**,**B**) and non-deformed polypropylene gel electrophoresis (**C**,**D**): (**A**,**C**) (a) H1; (b) H1 + H2; and (c) iDNA + H1 + H2; (**B**,**D**) (d) Cu^2+^-dependent DNAzyme + H0 + λ-Exo +H1 + H2; (e) Cu^2+^ + Cu^2+^-dependent DNAzyme + H0 + λ-Exo +H1 + H2; (f) Cu^2+^ + Cu^2+^-dependent DNAzyme + H0 + λ-Exo (little) + H1 + H2; and (j) Cu^2+^ + Cu^2+^-dependent DNAzyme + H0 + λ-Exo + H1. C_(Cu^2+^)_ = 10 pmol·L^−1^; C_(Cu^2+^-dependent DNAzyme)_ = 60 nmol·L^−1^; C_(λ-Exo)_ = 0.16 U/μL; C_(H0)_ = C(H1) = C(H2) = 100 nmol·L^−1^.

To verify the feasibility of the method, fluorescence spectroscopy was employed. To guarantee efficient detection with a high S/N, the background signal of MCHA was first determined. As shown in Figure 1A, the fluorescence intensity of only H1 (curve a) was almost the same as those of H1 and H2 (curve b). However, when iDNA (as the initiator) was introduced, MCHA was triggered, resulting in a strong fluorescent signal (curve c). This clearly confirmed the low background signal of MCHA and provided an effective guarantee for the cascade fluorescence reaction system.

Furthermore, as shown in Figure 1B, the effect of the cascade signal amplification strategy of this fluorescence biosensor was verified, including “recycle I” and “recycle II”. For “recycle I”, compared with sample a featuring a complete reaction system (curve e), sample b had less λ-Exo added to limit the initiation of MCHA. As expected, with the amount of λ-Exo reduced, the amplification efficiency was reduced, resulting in a relatively weak fluorescence intensity (curve f), which effectively verified the signal amplification effect of λ-Exo. For “recycle II”, the amplification effect of MCHA was proven by sample c, which only contained H1 of MCHA. Its fluorescence intensity (curve j) was obviously weaker than that of sample a (curve e). This demonstrated that H1 was hybridized, resulting in a fluorescent signal. However, due to the lack of H2, MCHA could not be triggered, which effectively verified the amplification effect of MCHA. The fluorescence spectral data demonstrated that the developed fluorescence biosensor could effectively detect Cu^2+^. Native polyacrylamide gel electrophoresis was first used to validate our Cu^2+^ detection strategy. As shown in Figure 1C, the combination of H1 and H2 showed a clear band (lanes a) at a similar location to H1 (lane b), showing that the H1 and H2 hairpin probes could coexist stably in the absence of the trigger chain. When trigger strand iDNA was present (lane c), CHA could be activated. As shown in Figure 1D, when Cu^2+^ was not present in the reaction system (lane d), the band mobility was much lower than when Cu^2+^ was present (lane e), showing that Cu^2+^ was effectively detected and initiated the subsequent reaction. The decrease in strip mobility when decreasing the amount of λ-Exo in the system (lane f) indicates that, as λ-Exo decreased, the trigger chain release decreased and thus the self-assembly efficiency decreased. CHA was not triggered when H2 (lane j) was not added to the reaction system.

### 3.3. Optimization of Experimental Conditions

To maximize the detection performance of the developed biosensor, the experimental conditions were cautiously optimized, including the MCHA and λ-Exo digestion reaction.

**Figure 2:**
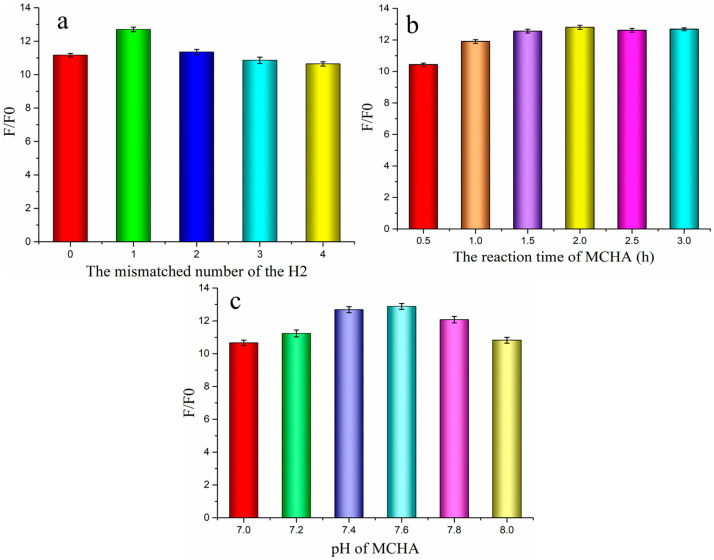
Optimization of experimental conditions: (**a**) mismatched number of H2; (**b**) reaction time of MCHA; and (**c**) pH of MCHA. The error bars show the standard deviations of three independent experiments.

As exhibited in the reaction mechanism, the fluorescent signal of the reaction system came from the MCHA. Furthermore, the MCHA S/N ratio could directly determine the sensitivity of the detection system. Therefore, MCHA was first optimized (Figure 2), including the mismatched number of H2, reaction time, and MCHA pH. As shown in Figure 2a, the S/N ratio reached the maximum at the mismatched number of 1; thus, “1” was chosen as the appropriate mismatched number. Subsequently, the MCHA reaction time was investigated. As shown in Figure 2b, the S/N ratio increased with an increase in the reaction time from 0 to 2.0 h, and a further temperature increase led to a decrease in the S/N ratio. Thus, 2.0 h was chosen for the subsequent test. In addition, the pH of MCHA is of vital importance for the sensitivity of MCHA. As shown in Figure 2c, the S/N gradually increased from 7.0 to 7.6, but the value reduced slowly thereafter. Hence, 7.6 was selected as the most appropriate pH.

**Figure 3:**
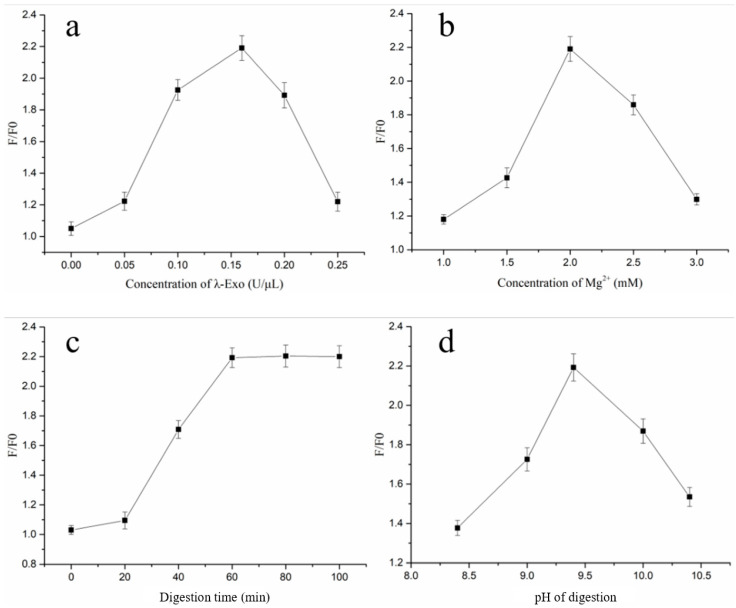
Optimization of experimental conditions: (**a**) λ-Exo concentration; (**b**) Mg^2+^ concentration; (**c**) digestion time; and (**d**) digestion reaction’s pH.

The λ-Exo digestion reaction was also carefully optimized, with respect to parameters including the λ-Exo concentration, Mg^2+^ concentration, digestion time, and digestion reaction’s pH. As shown in Figure 3a, the S/N ratio increased with an increase in the concentration of λ-Exo from 0 to 0.16 U/μL, but it decreased upon further increasing this concentration. The reason for this is that an excessive concentration of λ-Exo may interfere with its digestion reaction and lead to a nonspecific hydrolysis reaction mediated by λ-Exo, causing a significant decrease in the S/N ratio. Thus, 0.16 U/μL was selected for optimization. Subsequently, the Mg^2+^ concentration was optimized because of its crucial impact on the λ-Exo digestion reaction. As shown in Figure 3b, when the Mg^2+^ concentration in the λ-Exo digestion reaction was increased from 1.0 to 2.0 mmol·L^−1^, the S/N ratio gradually increased, but it decreased thereafter. Thus, 2.0 mmol·L^−1^ was chosen as the optimal Mg^2+^ concentration for the λ-Exo digestion reaction. In addition, the digestion time plays a considerable role in improving the optimization efficiency. As displayed in Figure 3c, the S/N ratio improved gradually with an increase in the digestion time, before stabilizing at 60 min. Thus, 60 min was selected as the optimum digestion time. Lastly, the λ-Exo digestion reaction’s pH was optimized, as shown in Figure 3d. The S/N ratio gradually increased with an increase in the pH from 8.5 to 9.4. However, with a further increase in the pH, the value decreased gradually. The reason for this may be that the λ-Exo activity was deactivated at a pH higher than 9.4. Therefore, 9.4 was chosen as the best pH for the digestion reaction.

### 3.4. Detection Performance of the Cu^2+^ Fluorescence Biosensor

**Figure 4:**
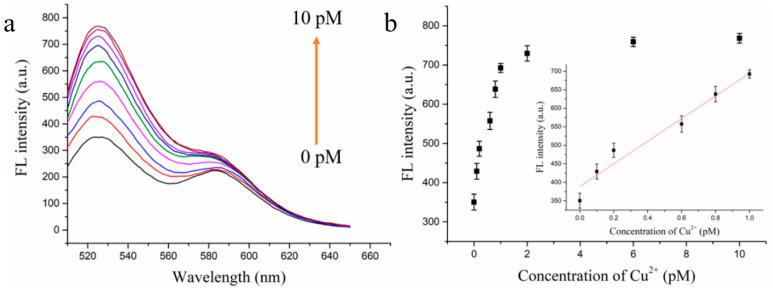
(**a**) The fluorescence response of the sensor at various concentrations of Cu^2+^: 0, 0.1, 0.2, 0.6, 0.8, 1, 2, 6, and 10 pM. (**b**) The linear regression equation diagram of this biosensor for various concentrations of Cu^2+^. The error bars show the standard deviations of three independent experiments.

To assess the performance of the developed biosensor, the sensitivity of the assay was evaluated under optimum experimental conditions. Accordingly, several samples with various concentrations of Cu^2+^ were analyzed, and the fluorescence emission spectra were recorded. As shown in Figure 4, the fluorescence intensity gradually increased with an increase in Cu^2+^ concentration (Figure 4a), exhibiting a strong linear relationship in the Cu^2+^ concentration range of 200 to 1000 fM. The corresponding linear regression equation was *y* = 310.84*C* + 383.83, and the correlation coefficient (*R*^2^) was 0.9682, where *y* is the fluorescence intensity and *C* is the concentration of Cu^2+^ (Figure 4b). According to 3σ/S (where σ is the standard deviation of blank sample and S is the slope of the calibration curve), the limit of detection (LOD) of the biosensor was 60 fM. In addition, we compared the detection performance of this biosensor with that of previously published Cu^2+^ fluorescence biosensors (Table S2). Despite requiring a relatively long assay time, the proposed biosensor in this paper is more sensitive, demonstrating a lower LOD than other approaches.

### 3.5. Specificity of the Cu^2+^ Fluorescence Biosensor

**Figure 5:**
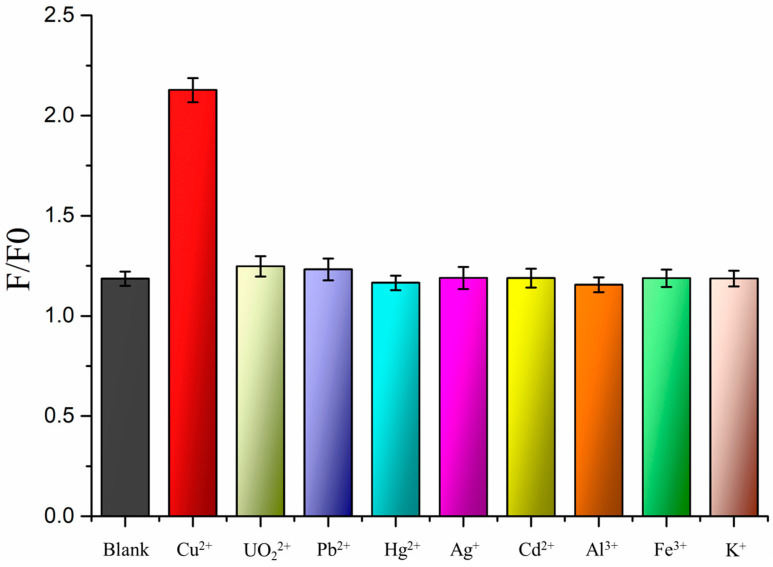
Selectivity of several other metal ions, including UO^22+^, Pb^2+^, Hg^2+^, Ag^+^, Cd^2+^, Al^3+^, Fe^3+^, and K^+^, at concentrations 50 times higher than the concentration of Cu^2+^.

To investigate the selectivity of this biosensor, several other metal ions, including UO_2_^2+^, Pb^2+^, Hg^2+^, Ag^+^, Cd^2+^, Al^3+^, Fe^3+^, and K^+^, at a concentration 50 times higher than the Cu^2+^ concentration were tested. As shown in Figure 5, only the target ion (Cu^2+^) exhibited a high S/N ratio, while the other metal ions displayed lower values. These results indicate that this biosensor possesses outstanding selectivity for Cu^2+^.

### 3.6. Detection of Cu^2+^ in Real Samples

To further demonstrate the possible application of the proposed biosensor for detecting Cu^2+^ in real samples, Xiangjiang river water collected from Hengyang, China, was tested. However, the signal intensity was limited for the detection of Cu^2+^ in the real samples. Therefore, we added different concentrations of Cu^2+^ standard solutions (400, 600, and 800 fM) to the real samples to recover the samples (Table 1).

## 4. Conclusions

In conclusion, an ultrasensitive DNAzyme-based fluorescence biosensor for detecting Cu^2+^ was successfully designed, featuring a cascade signal amplification strategy coupling λ-exonuclease-assisted target recycling and MCHA. Owing to the dual-signal amplification based on the cascade signal amplification strategy and the high S/N ratio based on MCHA, the fluorescent biosensor could detect Cu^2+^ efficiently, exhibiting a good linear range with an LOD of 60 fM. Furthermore, the method showed considerable selectivity toward Cu^2+^ with respect to other metal ions. Lastly, this assay exhibited a noteworthy performance in Xiangjiang river water. Therefore, the proposed biosensor with a cascade signal amplification strategy can be considered as a novel DNA circuit for detecting other targets with trace levels of Cu^2+^ in the future.

## Figures and Tables

**Table 1:** Detection of Cu^2+^ in Xiangjiang river water.

Xiangjiang River Water	Added (fM)	Found Mean ± SD *	Recovery (%)
Sample 1	400	425.55 ± 0.6	106.38
Sample 2	600	587.59 ± 0.4	97.93
Sample 3	800	808.20 ± 0.2	101.03

* SD, the standard derivation of three measurements.

## Data Availability

Not applicable.

## Supplementary Materials

The following supporting information can be downloaded at https://www.mdpi.com/article/10.3390/bios13100918/s1: Table S1. The oligonucleotide sequences used in this study; Table S2. A detailed comparison of different methods for detecting Cu^2+^. References [32,33,34,35,36,37,38,39,40,41] are cited in the supplementary materials.
