# Supplementary material for: A Cascade Signal Amplification Strategy for the Ultrasensitive Fluorescence Detection of Cu2+ via λ-Exonuclease-Assisted Target Recycling with Mismatched Catalytic Hairpin Assembly
Source: Biosensors (Basel). 2023 Oct 8;13(10):918. doi: 10.3390/bios13100918 (PMC10605925; doi:10.3390/bios13100918)
Supplement: Supplementary file 1 [file biosensors-13-00918-s001.zip › biosensors-2494852-supplementary.pdf]

Supplementary Information

A Cascade Signal Amplification Strategy for the Ultrasensitive Fluorescence Detection of Cu^{2+} via λ-Exonuclease-Assisted Target Recycling with Mismatched Catalytic Hairpin Assembly

Zhen Liu [1], Chen Liu [2], Liqiong He [1], Jinquan Liu [1,*], Le Li [1], Shengyuan Yang [1], Yan Tan [1], Xing Liu [1] and Xilin Xiao [1,3,*]

[1] Hunan Key Laboratory of Typical Environmental Pollution and Health Hazards, School of Public Health, Hengyang Medical School, University of South China, Hengyang 421001, China; m13677341048@163.com (Z.L.); hlq_joan@163.com (L.H.); usclile@126.com (L.L.); yangshyhy@126.com (S.Y.); tanyan007@163.com (Y.T.); 18206767749@163.com (X.L.)

[2] Hunan Province Key Laboratory for Typical Environmental Pollution and Health Hazards, School of Chemistry and Chemical Engineering, University of South China, Hengyang 421001, China; liuchen5573@163.com

[3] State Key Laboratory of Chemo & Biosensing and Chemometrics, Hunan University, Changsha 410082, China

* Correspondence: letliufly@163.com (J.L.); xiaoxl2001@163.com (X.X.)

Materials and methods

Materials and reagents

Table S1. The oligonucleotide sequences used in this study.

Oligonucleotide	Sequence 5′-3′
E-DNA	GGT AAG CCT GGG CCT CTT TCT TTT TAA GAA AGA AC
S-DNA	CTT CTT TCT AAT ACG GCT TAC CA
H0	PO_4-GTG GTA AGC ATA GTG AGT GAC CTT TTC ATC CCT CTG TAG AAA AGG TCA CTC ACT ATG
H1	ATA GTG AGT GAC C**T**(TAMRA)T TTC TAC CAC CAG ACT ACG GTA GAA AAG G**T**(FAM)C ACT GT
H2(0)	TCT ACC GTA GTC TGG TGG TAG AAA AGG TAC CAG ACT AC
H2(1)	TCT ACC GTA GTC TGG TGG TAG AAA AGG AAC CAG ACT AC
H2(2)	TCT ACC GTA GTC TGG TGG TAG AAA AGA AAC CAG ACT AC
H2(3)	TCT ACC GTA GTC TGG TGG TAG AAA ATA AAC CAG ACT AC
H2(4)	TCT ACC GTA GTC TGG TGG TAG AAA TTA AAC CAG ACT AC

Preparation of Cu^{2+}-dependent DNAzyme and hairpin DNA

E-DNA (10.0 μmol L^{-1}) and S-DNA (10.0 μmol L^{-1}) were mixed in equal volumes, forming the Cu^{2+}-dependent DNAzyme preparation solution (5.0 μmol L^{-1}). Subsequently, the mixture was heated to 95 °C for 7 min for annealing and then cooled slowly to room temperature (25 °C) to form stable Cu^{2+}-dependent DNAzyme.

The hairpin DNAs (10.0 μmol L^{-1}), including H0 labelled with a phosphate group at the 5-terminus, H1 labelled with FAM and TAMRA and H2 labelled with no group, were respectively heated to 95 °C for 7 min for annealing and then cooled slowly to room temperature (25 °C) to form firm hairpin structure.

The prepared Cu^{2+}-dependent DNAzyme and the hairpin DNAs can be stored in the refrigerator (4 °C) before use.

Apparatus

Fluorescence spectrometer

The voltage was 700 V, the excitation wavelength was set to 485 nm, the excitation slit and the emission slit were both 5 nm, and the scanning speed was 1200 nm / minute. Under these conditions, emission spectra in the range of 500–650 nm were collected, and the fluorescence intensity at 525 nm was used as the basis for evaluating the analytical performance of the proposed biosensor.

PAGE characterization

PAGE was used to demonstrate the feasibility of the proposed detection system consisting of λ Exo-assisted target recycling and MCHA. 5 μL of the reacted sample was added to 2 μL of 10 × SYBR Green I and then reacted for 5 min in the dark. Subsequently, the mixture was added to the freshly prepared 15% polyacrylamide gel and electrophoresed in 1 × TBE buffer (89 mM Tris-boric acid, 2 mM EDTA, pH 8.2) at a constant voltage of 110 V for 90 min.

Supporting Table

Table S2. Detailed comparison of different methods for detecting Cu^{2+}.

Sensing method	Detection technique	Linear range	LOD	Year	Ref.
DNAzyme + SG	Fluorescence	40 nM-1200 nM	10 nM	2013	S1
AuNPs + Cu^{+}-catalyzed click chemistry	Lateral flow biosensor	50 nM-200 μM	100 nM	2015	S2
HCR + MB+SG	Fluorescence	20 pM-1μM	12.8 pM	2013	S3
HCR + FRET + MB	Fluorescence	0-100 nM	0.5 nM	2016	S4
EDC	Colorimetry	0-120 nM	1.3 nM	2017	S5
HCR + triplex DNA	Fluorescence	5.7 nM-455 nM	2 nM	2019	S6
DNAzyme	Personal glucose meters	-	1 nM	2017	S7
ExoIII + ExoI + Cu^{+}-catalyzed click chemistry	Fluorescence	0-10 μM	39 nM	2020	S8
AuNPs + Cu^{+}-catalyzed click	Colorimetry	1 nM-100 uM	26.8 nM	2021	S9
Ni/Fe layered double hydroxide nanosheet + MB	Electrochemical	1 pM-10 uM	0.29 pM	2022	S10
λ-Exo + MCHA	Fluorescence	200 fM-1000 fM	60 fM	-	Our method

References:

[S1] L.L. Zhang, Y.Y. Zhang, M.J. Wei, Y.H. Yi, H.T. Li, S.Z. Yao, A label-free fluorescent molecular switch for Cu^{2+} based on metal ion-triggered DNA-cleaving DNAzyme and DNA intercalator, New J. Chem. 37 (2013) 1252-1257, https://doi.org/10.1039/C3NJ41103F.

[S2] D. Wang, C.C. Ge, L. Wang, X.R. Xing, L.W. Zeng, A simple lateral flow biosensor for the rapid detection of copper (II) ions based on click chemistry, RSC. Adv. 5 (2015) 75722-75727, 10.1016/j.aca.2014.12.041.

[S3] C.C. Ge, J.H. Chen, W. Wu, Z.Y. Fang, L.B. Chen, Q. Liu, et al., An enzyme-free and label-free assay for copper (II) ion detection based on self-assembled DNA concatamers and Sybr Green I, Analyst. 138 (2013) 4737-4740, https://doi.org/10.1039/C3AN00973D.

[S4] Y. Chen, L. Chen, Y.D. Ou, Z.H. Wang, F.F. Fu, L.Q. Guo, DNAzyme-based biosensor for Cu^{2+} ion by combining hybridization chain reaction with fluorescence resonance energy transfer technique, Talanta. 155 (2016) 245-249, https://doi.org/10.1016/j.talanta.2016.04.057.

[S5] Y. Park, C.Y. Lee, K.S. Park, H.G. Park, Enzyme-Free Colorimetric Detection of Cu^{2+} by Utilizing Target-Triggered DNAzymes and Toehold-Mediated DNA Strand Displacement Events, Chem-Eur J. 23 (2017) 17379-17383, https://doi.org/10.1002/chem.201704346.

[S6] R.D. Shen, L. Zou, S.X. Wu, T.T. Li, J. Wang, J.M. Liu, et al., A novel label-free fluorescent detection of histidine based upon Cu^{2+}-specific DNAzyme and hybridization chain reaction, Spectrochim. Acta A. 213 (2019) 42-47, https://doi.org/10.1016/j.saa.2019.01.062.

[S7] J.J. Ming, W.B. Fan, T.F. Jiang, Y.H. Wang, Z.H. Lv, Portable and sensitive detection of copper (II) ion based on personal glucose meters and a ligation DNAzyme releasing strategy, Sens. Actuators, B-Chem. 240 (2017) 1091-1098, https://doi.org/10.1016/j.snb.2016.09.091.

[S8] Y.F. Qin, M. Li, Y.Y. Yang, Z.Y. Gao, H.S. Zhang, J.J. Zhao, A unimolecular DNA fluorescent probe for determination of copper ions based on click chemistry, RSC. Adv. 10 (2020) 6017-6021, 10.1039/C9RA10174H.

[S9] W.C. Yan, Z.S. Zhong, J. Ma, T. Rujiralai, Highly sensitive colorimetric sensing of copper (ii) ions based on "CLICK-17" DNAzyme-catalyzed azide modified gold nanoparticles and alkyne capped dsDNA cycloaddition, RSC. Adv. 11 (2021) 24196-24205, 10.1039/D1RA03813C.

[S10] A. Szerlauth, L. Szalma, S. Muráth, S. Sáringer, G. Varga, L. Li, et al., Nanoclay-based sensor composites for the facile detection of molecular antioxidants, Analyst. 147 (2022) 1367-1374, 10.1039/D1AN02352G.
